# “Rolling the Boulder Up the Hill”: A Qualitative Study of Parents’ Experiences Providing Ongoing Care at Home for Their Child with Complex Medical Needs

**DOI:** 10.1177/08445621251380909

**Published:** 2025-10-16

**Authors:** Joanne Tay, Adam Rapoport, Jamie Crawley, Joanne Ta, Jessica C. Kichler

**Affiliations:** 1 Faculty of Nursing, 8637 University of Windsor, Windsor, ON, Canada; 2 Emily’s House Children’s Hospice, Toronto, ON, Canada; 3 Paediatric Advanced Care Team, 4956 The Hospital for Sick Children, Toronto, ON, Canada; 4 Departments of Paediatrics and Family & Community Medicine, University of Toronto, Toronto, ON, Canada; 5 Faculty of Arts, Humanities and Social Sciences, Department of Psychology, 8637 University of Windsor, Windsor, ON, Canada

**Keywords:** Caregiver, children and young people, complex care, mental health, qualitative approaches

## Abstract

**Background & Purpose:**

Parents of children with medical complexity (CMC) provide continuous, intensive care that encompasses a range of technical tasks, emotional support, advocacy, and coordination within fragmented healthcare systems. Existing research often treats caregiving as a discrete phenomenon, overlooking how parents make meaning of their roles amid uncertainty and moral distress. This study aimed to explore the lived experiences of parents caring for children with medical complexity at home, examining how caregiving is experienced, sustained, and made meaningful in everyday life.

**Methods & Procedures:**

An exploratory qualitative design was used to conduct in-depth, semi-structured interviews with 15 parents (13 mothers, two fathers) of CMC receiving home care in Ontario, Canada. Purposive sampling was used to recruit parents. Data were analyzed using Braun and Clarke's thematic analysis approach.

**Results:**

Two overarching themes emerged from parents’ accounts: (1) The Layered and Relentless Nature of Caregiving; and (2) The Transformation of Self Through Caregiving, with effects spanning social life, identity loss, mental and physical health decline, and financial strain. Parents reported role overload, identity loss, chronic fatigue, emotional isolation, and ongoing pressure to advocate within unresponsive systems. The lack of consistent and reliable home care and financial support intensified challenges.

**Conclusion:**

Caring for a CMC impacts parental well-being and reshapes their identity. Sustainable caregiving requires policies and services that support more than just childcare. Integrated mental health services, equitable access to respite, income protections, and caregiver-informed systems are needed to relieve families of unsupported responsibilities and ensure the long-term sustainability of home-based complex care.

## Background

A child with medical complexity (CMC) is broadly defined as having medical fragility and intensive care needs, often due to congenital or acquired multisystem diseases, severe neurological conditions, or technology dependence (e.g., feeding tube, wheelchair, assistive breathing device) for daily living activities (Cohen et al., 2011). These children require continuous, highly technical, and individualized care for multiple chronic conditions, extending beyond physical care tasks to include coordinating medical appointments, managing specialized equipment, and advocating across fragmented healthcare systems (Hirt et al., 2023; Lattanza et al., 2024; Miller et al., 2009; Rempel & Scott, 2014).

Despite increasing recognition of caregiver burden, most research focuses on discrete and measurable outcomes such as psychological distress, navigation difficulties, and unmet needs (Bayer et al., 2021; Costa et al., 2024; Cousino & Hazen, 2013; Lindström et al., 2010; Prieto et al., 2022; Schnell et al., 2025). These approaches typically conceptualize burden through static measures, such as standardized scales, stress assessments, and task inventories, which quantify caregiving difficulties at a single point in time. While studies focusing on discrete and measurable outcomes have advanced understanding of mental health risks and systemic barriers, they often rely on cross-sectional data and standardized measures that fail to capture how caregiving is lived, interpreted, and sustained over time. As a result, caregiving is frequently framed in narrow terms, reduced to levels of burden or instrumental needs like respite or training, while overlooking its deeper emotional, identity-related, and moral dimensions.

Emerging qualitative research has begun to explore these underexamined layers, highlighting experiences such as emotional erosion of self (Teicher et al., 2023), identity loss and constant vigilance (i.e., the need for continuous monitoring and readiness to respond to medical crises) (Currie & Szabo, 2020; Salami & Alhalal, 2024; Smith et al., 2022), and the fragmentation of caregivers’ social and professional lives (Brenner et al., 2021; Chung et al., 2013; Hirt et al., 2023). Yet, significant gaps remain in understanding the relational and often ambivalent nature of caregiving, particularly how caregivers experience, sustain, and make meaning of their roles in everyday life amid uncertainty and competing demands.

This study addresses these gaps by centering the lived experiences of those caring for CMC at home. Within this study, “parents” refers to primary caregivers of CMCs, whether biological relatives, guardians, or others in a parental role. In Ontario, families access home care through government-funded agencies and private providers, with some opting for family-managed care models, where parents assume responsibility for hiring and managing care staff. Despite these options, many caregivers are left to coordinate and deliver complex care without sufficient training or formal support, increasing anxiety about care quality and reliability while compounding emotional and psychological strain.

This study draws on Pearlin and colleagues’ Caregiving Stress Process Model (Pearlin et al., 1981, 1990), which conceptualizes caregiving as a complex, dynamic process. Moreover, this framework encompasses primary stressors (directly related to the child's condition), secondary stressors (arising from the caregiving role itself), and various outcomes that affect caregiver well-being (Pearlin et al., 1981, 1990; Raina et al., 2004). This framework enables a more comprehensive understanding of how caregiving demands can permeate multiple life domains, aligning with our focus on how caregiving is experienced and sustained in everyday life.

By foregrounding caregivers’ perspectives, this study aimed to explore the lived experiences of parents caring for children with medical complexity at home, examining how caregiving is experienced, sustained, and made meaningful in everyday life. Rather than viewing caregiving as a mere set of tasks or measurable burden, this study conceptualizes caregiving as an ongoing relationship between parent and child that unfolds through daily interactions, physical care activities, and the emotional work of navigating uncertainty. In doing so, it offers critical insight into the factors shaping the sustainability of care at home and informs policies, services, and systems aimed at supporting caregivers and advancing equitable, responsive pediatric home care.

## Methods

An exploratory qualitative design was used to generate an in-depth understanding of a phenomenon that has been inadequately examined in existing literature. This approach was well-suited to our study because it allows researchers to investigate complex social processes and meaning-making experiences without imposing a predetermined theoretical framework, enabling the emergence of insights directly from participants’ accounts of their caregiving realities.

### Recruitment and Participants

Participants were recruited from pediatric community care organizations within Ontario using purposive and snowball sampling strategies through social media and email. Eligibility criteria included English-speaking parents or primary caregivers of children aged 0–18 years with chronic neurological impairment and/or multisystem condition, technology dependence, and follow-up by either a children's complex care team or a minimum of three children's subspecialists. Participants must have received or previously received pediatric home care services (e.g., nursing support, personal care assistance, medical equipment management) in Ontario and have access to Microsoft Teams. Parents of children receiving active end-of-life care were excluded.

### Ethical Consideration

Following approval by the University of Windsor Research Ethics Board (Reference Number: 24–113), all participants provided written informed consent via REDCap. Each participating organization granted permission to recruit and disseminate study information but did not participate in data collection. Given the potentially distressing nature of discussing caregiving challenges, participants were provided with detailed information about the study up front and given time to consider their participation. At the beginning of each interview, participants were reminded of their right to withdraw at any point during the interview or to skip any interview questions that caused discomfort. Prior to the interview, each participant received a copy of the electronic consent form and a digital brochure listing local and provincial mental health resources, including free counselling/mental health services. Interviewers also checked in with participants throughout the interview process to ensure they were not experiencing undue emotional distress. Each participant received a $$25 gift card as an appreciation for their time.

### Data Collection

Two nurse researchers conducted semi-structured interviews, one with pediatric home care experience and the other a recent graduate with qualitative research training. Interviews lasted between 45 and 90 min, with an average duration of approximately 60 min. Before each session, participants completed a demographic questionnaire. The interview guide explored participants’ daily caregiving experiences, emotional responses to caregiving demands, interactions with healthcare systems, perceived barriers and facilitators to care, impacts on family relationships and personal identity, and recommendations for service improvement (see Supplementary Table 1). All interviews were conducted virtually via Microsoft Teams, audio-recorded with consent, and transcribed verbatim. Transcripts were anonymized, de-identified, and reviewed for accuracy. Data were stored on a secure, password-protected server at the University of Windsor, accessible only to the research team.

### Data Analysis and Interpretation

Data were analyzed using reflexive thematic analysis, which offers flexibility in identifying, analyzing, and interpreting themes (Braun & Clarke, 2021). This reflexive approach was well-suited to exploring the subjective, lived experiences of caregivers, as it recognizes that themes are actively constructed through the researchers’ engagement with the data rather than discovered. The method's emphasis on researcher reflexivity was significant given our team's insider knowledge of pediatric complex care. De-identified transcripts were uploaded to NVivo to support analysis. Following Braun and Clarke's (2021) iterative approach, transcripts were reviewed, coded, discussed, and grouped into themes and subthemes. In the familiarization phase (Step 1), both researchers independently read and reread the transcripts, recording their initial analytic impressions. During coding (Step 2), statements were highlighted to identify patterns, with initial coding guided by the research questions and emerging inductively from the data, including patterns related to caregiving roles, access barriers, identity, emotional burden, and systemic challenges. Initial codes were clustered into tentative themes through discussion (Step 3), and the team reviewed these to ensure alignment with participants’ experiences. Themes and subthemes were refined to ensure coherence and distinction (Steps 4 and 5). In the final phase (Step 6), themes were organized into key caregiving experiences and supported with rich NVivo quotations to preserve narrative depth and context.

### Trustworthiness

We employed several strategies to enhance trustworthiness and credibility, including methodological congruence, audit trail, peer debriefing, and reflexive journaling (Braun & Clarke, 2021). Two researchers contributed to the data analysis, maintaining audit trails of analytic impressions to ensure that the interpretation was grounded in the data. We met frequently to debrief emerging insights and ensure interpretations remained grounded in participant experiences. As researchers and healthcare providers with insider knowledge of CMC, we acknowledged our positionalities and maintained active, reflexive stances during data collection and analysis to ground our preconceived notions in participant experiences.

## Results

Of the 22 individuals who completed the screening form, 15 participants (13 mothers and two fathers) were selected for interviews. Table 1 presents an overview of participant demographics, including income levels, employment status, and utilization of home care services. Through our reflexive thematic analysis, we developed two overarching themes: 1) The Layered and Relentless Nature of Caregiving, and 2) The Transformation of Self Through Caregiving. From Theme 1, three subthemes were derived: a) layered caregiving responsibilities, b) hidden labour of advocacy, and c) constant search for caregiving support. From Theme 2, five subthemes emerged: a) social strain of sustained caregiving, b) erosion of personal identity through medicalized parenting, c) cumulative mental health impact of constant responsibility, d) physical strain from unrelenting round-the-clock care, and e) financial pressures of unpaid care. Each theme was comprised of several subthemes that illustrate the depth and diversity of participants’ experiences across multiple life domains.

**Table 1.** Demographic Characteristics of Parents (N = 15).

Characteristics	Value
**Age, years**	Mean = 41, Range = 30–52
**Duration of home care access, years**	Mean = 5, Range = 0.5–10
**Relationship to child with medical complexity (CMC)**	
Father	2
Mother	13
**Number of children (including CMC)**	
One	11
Two	2
Three	2
**Employment status**	
Full-time	3
Part-time	7
Self-employed	3
Not employed	2
**Family income**	
Under $$25,000	2
$$25,000 - $$49,999	1
$$50,000 - $$99,999	7
$$100,000 or more	3
Prefer not to say	2
**Home care services used**	
Nurse	11
Personal Support Worker (PSW)	8
Physiotherapist (PT)	4
Occupational Therapist (OT)	6
Speech-language Therapist (SLT)	4
Dietician	2
**Need for overnight care for CMCs**	
Yes	10
No	5

*Note.* CMC = child with medical complexity.

### The Layered and Relentless Nature of Caregiving

This theme captures the expansive and relentless responsibilities parents of CMC assume for their child's physical, emotional, developmental, and social well-being. Their lives were defined by the demands of caregiving, often requiring them to assume multiple roles to provide high levels of instrumental support for their children. This overarching theme was comprised of the following three subthemes: 1) layered caregiving responsibilities, 2) hidden layer of advocacy, and 3) constant search for caregiving support.

#### Layered Caregiving Responsibilities

Nearly all participants (n = 14) described the overwhelming nature of performing multiple, complex roles within the home, including those of nurse, therapist, care coordinator, and medical decision-maker, in addition to being a parent. These roles were not optional but emerged out of necessity in response to inconsistent home care, unresponsive systems, and a lack of viable alternatives. “Overwhelming” was a recurring descriptor, as parents attempted to balance care responsibilities with the emotional demands of parenting. One participant captured the impossible trade-offs forced upon them:

…I had the choice, let my kid die because the system can’t support me, or give it a go and see how things play out…yes, it's a decision of the parents, but it's informed by something… Some of that is outside the hands of the parents in that situation making moments (Participant 7).

Participants described how they assumed multiple roles within the home, including tasks such as suctioning, administering medication, and monitoring vital signs. Many spoke about the tension between their parenting role and the medical care responsibilities they were required to perform. As one parent explained, this shift fundamentally reshapes the parent–child relationship:

You're their comfort person and you're also the one putting in their catheter. Like, that's a hard line to walk. (Participant 12).

These blurred boundaries often manifested as role conflict. Caregivers wrestled with the emotional toll of being perceived by their child not as a source of comfort but as a source of procedures and clinical demands. Some sought to resist this shift by preserving nurturing routines such as cuddling before bed, preparing favourite foods, and engaging in play. However, maintaining these moments of connection required extra effort and attention, especially amid the emotional strain and competing demands of care.

The strain of assuming multiple roles was compounded in families with more than one child. Many participants (n = 9) described feeling deep guilt about the unequal distribution of their time and energy. As one caregiver acknowledged, “*I think we were in survival, and my older daughter had a lot of neglect. So, a lot of guilt for that*” (Participant 13). This sense of guilt was not abstract. It was entangled in everyday realities. Caregivers made deliberate efforts to shield siblings from the burdens of caregiving, refusing to shift responsibilities onto them despite the need for help:

People say, ‘oh, siblings can help.’ That's not their purpose. They are a child; they should flourish and grow and get to do all the things that children do… They shouldn’t have to worry about trying to help their other sibling (Participant 12).

While some respondents found creative ways to co-parent, share responsibilities, or utilize extended family support, the overarching narrative was one of parental role overload. For many (n = 12), caregiving became an all-consuming identity, rendered necessary by a system that too often failed to deliver consistent, qualified home care. This left families in a precarious position, managing not just their child's medical needs but also their emotional survival.

#### Hidden Labour of Advocacy

Many caregivers described the exhausting and often invisible burden of advocacy. Navigating the provincial home care system was usually described as fragmented and unclear, requiring parents to both learn and challenge it, question decisions, request reassessments, and correct misinformation about their child's care. Advocacy was not a choice but a necessity, often the only means by which caregivers could secure the services that their children were entitled to.

One parent recalled how a case coordinator initially dismissed their request for nursing, citing a narrow interpretation of eligibility criteria:

When I contacted [the care coordinator] and asked about funding, she said, ‘oh, [your child] wouldn’t qualify. [Your child] only has a G-tube.’ But when I looked at the website, it said, ‘for G-tube feeds, you should qualify’ (Participant 13).

Had this caregiver accepted that response from the case coordinator at face value, their child may have gone without essential care. Instead, they relied on information shared by other parents to challenge the initial assessment:

If I had said, ‘oh, okay,’ and hung up, I still wouldn’t have it to this date. But because I knew from that Facebook group… other people had said, ‘my child has a G-tube and they get nursing,’ then I knew I had to keep pushing… so that's advocating (Participant 13).

This account illustrates how formal pathways to care are often obstructed by gatekeeping, misinformation, or inadequate training of service coordinators. In response, many caregivers turned to informal support networks, such as online groups, peer parents, and/or health professionals they knew personally, to gather critical information and validate their claims for their child with complex medical needs, in order to receive care. These networks became survival tools in an otherwise convoluted system.

Advocacy also involved seeking alternatives to mainstream service delivery. Several parents described opting into the Family-Managed Home Care program as a strategy to regain autonomy and consistency. Families could better address service gaps and avoid the unpredictability of agency-based care by directly hiring and training their staff. Yet even this solution came with significant administrative demands, including hiring, training, scheduling, and managing payroll, which parents were expected to undertake without formal training. One parent described it as “*almost like a full-time job managing his care,*” noting that “*on top of the nursing and the emotional toll and being a parent, there's all the coordination of services, the paperwork, the funding applications, it's a lot*” (Participant 9). For many, caregiving extended far beyond daily care tasks to include “*case management, advocacy, [and] accounting,*” (Participant 7) roles for which they struggled to find adequate support or recognition.

Across interviews, advocating for care was described as time-consuming, emotionally draining, and deeply frustrating. Caregivers spoke of delayed services, shifting criteria, and the constant threat of service reductions due to system reorganization or staffing shortages. Despite relentless efforts, many felt their concerns were deprioritized or disregarded:

You don’t always get somebody who knows what they’re doing. We would sometimes just let them do what they would do and then redo everything after they left (Participant 14).

Rather than being supported, families were routinely positioned as the mechanism through which access to care was achieved, rendering advocacy not as empowerment, but as compensatory labour for system shortfalls.

#### Constant Search for Caregiving Support

Many caregivers (n = 11) emphasized that the intensity of caring for a CMC could not be sustained without additional support. Despite this, most found themselves managing substantial responsibilities alone, often without adequate formal support. Caregivers described piecing together combinations of informal caregiving networks, such as partners, aging parents, extended family, neighbours, and online parent communities, to fill persistent gaps in care for their CMC. However, these arrangements were often described as burdensome and/or temporary, driven more by necessity than by preference, by outside support.

One caregiver described how their own parent had to retire early to meet basic caregiving needs due to prolonged shortages in home care nursing:

We were just sort of forced into this situation because we didn’t have enough home care nursing for a long time (Participant 2).

Others went even further, restructuring their lives entirely to gain access to this informal support. One parent reported giving up their career and moving back in with their parents to receive full-time help:

[I] moved back in with my parents… just to be able to manage it all, because [my child] needs 24/7 monitoring (Participant 8).

Outside extended family support, some caregivers described leaning on neighbours for help with basic care tasks, such as transferring a child into a vehicle or monitoring them briefly so the caregiver could step away. These acts of support, though deeply appreciated, also revealed the vulnerability of relying on informal, ad hoc systems to meet complex medical needs.

Many participants also pointed to social media and parent-to-parent networks as vital sources of information, emotional support, and practical guidance. Unlike formal healthcare channels, these peer connections were consistently cited as the most reliable and responsive form of knowledge exchange:

Only hearing about these resources from other parents that you’ve connected to… that's really where we get the best information. We get it not from the system but from other parents who have dealt with these problems and found solutions (Participant 11).

Some caregivers also utilized formal respite programs, which offered short-term relief, allowing them to rest or attend to other responsibilities. However, even these resources were often reported to be unevenly distributed and difficult to access. Geographical distance and logistical barriers usually rendered respite impractical or inaccessible, particularly for rural families:

It's more like five to six hours there, five to six hours to get him back again… It's easily 15 h of driving for a weekend getaway. Who's going to do that?… We’ve done it once or twice, and it was so exhausting to access it that we haven’t really accessed it since (Participant 9).

Caregivers made it clear that while supports exist, they are often fragmented, hard to access, or rely on personal networks and self-advocacy. The result is a fragile patchwork of care, built on exhaustion, sacrifice, and the goodwill of others, rather than a coordinated system designed to support families of children with complex medical needs. Collectively, the layered responsibilities, advocacy burden, and ongoing search for support demonstrate how caring for children with medical complexity requires parents to assume multiple roles while navigating inadequate systems, creating unsustainable demands that reshape family life and parental identity.

### The Transformation of Self Through Caregiving

Caring for a child with medical complexity profoundly transformed parents’ lives with relentless demands that overshadow social needs, identity, personal mental and physical health, and socio-economic security, rendering caregiving an all-consuming and often isolating experience. This overarching theme was comprised of the following subthemes: 1) social strain of sustained caregiving, 2) erosion of personal identity through medicalized parenting, 3) cumulative mental health impact of constant responsibility, 4) physical strain from unrelenting round-the-clock care, and 5) financial pressures of unpaid care.

#### Social Strain of Sustained Caregiving

Parents described a sense of social isolation that extended beyond the physical confines of the home. Many participants spoke of how difficult it was to find others who understood the specific challenges of raising a child with medical complexity. Even within the broader disability community, participants felt that their experiences were often too specialized or rare to be easily understood. Some found partial relief in online networks, particularly condition-specific Facebook groups, which served as both information hubs and social support:

We have a group for neurodiverse kids, and I have learned so much from these other parents… But it was really hard to find my community, and hang on to it… People with kids older than you will be happy to share their experience (Participant 14).

However, many noted that while these virtual spaces offered practical advice and validation, they were not a substitute for in-person social connection. The physical distance and the lack of condition-specific support groups meant that many caregivers still felt alone in their experiences.

This sense of isolation was often compounded by fear of misunderstanding or judgment from others, especially in public settings or institutional environments such as schools. Some participants recounted withdrawing from such spaces altogether to protect their child from stigma or misinterpretation:

[My child] is not able to get the care I think [my child] deserves at school… If another kid were to walk by and [my child] reached out to grab their shirt, [my child] wouldn’t be able to let go. So that's going to be a situation, and I don’t want [my child] to get labelled with behaviour issue… (Participant 14).

These concerns reflect the decisions parents undertake not only to protect their child's safety, but also their dignity, often in the face of systems that conflate behavioural difference with aggression or blame. The result is a “protective isolation,” where families remove themselves from social settings not out of preference, but self-preservation.

This withdrawal from potential social support networks and chronic stress also shaped intimate relationships. Parents described how the impact of caregiving for CMC spilled over into their partnerships, leaving little time or energy for connection. Some couples divided roles, one as caregiver, the other as breadwinner, but this often came at a cost:

…All they do all day long is attend to their kids’ medical needs, and their mental health plummets… Their marriages are being destroyed… And then this resentment builds between the couple, and the relationship falls apart, and the kids suffer because the parents are suffering (Participant 7).

Caregiving is physically demanding and destabilizes family dynamics, with tensions arising from structural pressures like inadequate support, as seen in these interviews. The social toll extends across relationships, leading to disconnect, guilt, resentment, and exhaustion, which erode caregivers’ well-being and sense of self.

#### Erosion of Personal Identity Through Medicalized Parenting

For many caregivers, the demands of caring for a CMC have reshaped their identity, with caregiving becoming the primary role that has displaced other aspects of themselves, such as professional, social, or personal identities. Caregivers described how career aspirations, educational goals, and daily routines were subordinated to the unpredictable nature of care for a CMC. While these life-altering decisions were often made out of love and necessity, they also came at a cost: a disconnection from their former selves. One participant, reflecting on the loss of their nursing career, offered a vivid account of this identity disruption:

So, I resigned from my job… my passion was to be a [healthcare provider]. Well, you can’t be a [healthcare provider] when you can’t guarantee you will be able to stay every day… I hate myself. I hate everybody. I can’t do it. So, I lost my pension and my career over 23 years. [I] kind of lost my identity. I always prided myself on being a really good [healthcare provider] (Participant 9).

This quote reflects the material consequences of leaving a profession and the emotional devastation of losing a role that once defined one's value and pride. For some, this identity loss extended beyond work to encompass a more profound sense of loss. Another participant highlighted how caregiving responsibilities for a CMC, when shouldered without adequate support, can consume a parent's entire identity:

I became a full-time caregiver, not by choice… my marriage is on the brink of being destroyed… my other kids suffer because we are suffering (Participant 7).

In this account, identity loss is framed not just as a personal experience but as an interpersonal issue, where caregiving becomes all-encompassing in the absence of sufficient external supports, with consequences that ripple through relationships and family dynamics.

Beyond the workplace, caregivers also described how caregiving encroached on the routines and roles that once gave meaning to daily life, such as friendships, hobbies, and time alone. The repetitive nature of medical caregiving, combined with isolation and exhaustion, led one parent to describe themselves as “*just a shell*,” noting, “*I didn’t even recognize myself anymore*” (Participant 9). These experiences were not marked by a single moment of rupture, but by a gradual unravelling of self. The accumulation of sacrifices such as missed milestones, altered relationships, and deferred aspirations gave way to feelings of invisibility, frustration, and grief for the loss of their former identity.

Compounding this erosion was the lack of recognition. Unlike professional roles, caregiving was seen as invisible labour, unacknowledged and often taken for granted. Caregivers seldom received validation for their sacrifices, nor were they offered meaningful ways to preserve aspects of their former identity:

At least if I were a shift nurse in a hospital, I would get paid, right? I would get breaks. I would be told, ‘here's an hour, go and take it.’ I don’t have that luxury (Participant 12).

The lack of validation deepened the emotional toll, as caregivers struggled to meet care demands while reconciling them with the loss of their former selves. Without support or recognition, they gradually faded from roles that once defined their identity, leaving an identity shaped by caregiving and marked by emotional sacrifice and loss.

#### Cumulative Mental Health Impact of Constant Responsibility

Caregivers described emotional depletion, with the relentless demands of CMC caregiving causing chronic strain and feeling overwhelmed. One caregiver spoke of reaching an emotional breaking point:

I needed to take a break and leave sometimes. But then I know when I’m leaving, I leave my [partner] alone… But I needed a break, or it would make me snap. So, it's like you’re constantly trying to balance all those needs (Participant 11).

This quote illustrates many caregivers’ emotional bind, a conflict between self-preservation and the fear of burdening others. The toll is internalized and relational, with caregivers often feeling that their need for rest comes at a cost to someone else's well-being. For some, the psychological strain extended beyond stress into trauma. The early period following diagnosis and hospital discharge was described as disorienting and emotionally destabilizing. One parent, reflecting on the long periods of adaptation to caregiving for a CMC, shared:

I did it all on my own, and I was wearing thin… It took a long time, from a personal standpoint, to get past that overwhelmed stage… and then after that, doing my work of processing trauma and dealing with issues related to PTSD (Participant 15).

Many parents described their distress beyond typical stress or exhaustion. Instead, many caregivers spoke of experiences that aligned more closely with symptoms of trauma, such as hypervigilance, emotional numbing, and difficulty returning to any sense of psychological baseline. Yet these symptoms often went unrecognized or unsupported. The cumulative weight of emotional, physical, and systemic pressures sometimes pushed caregivers to the edge of their capacity. Some parents described profound despair and hopelessness, experiences that, while not constituting active suicidal ideation, reflected the severe toll of sustained caregiving. One participant expressed this feeling with stark honesty:

If they asked me, then how I felt and if I thought I’d be better off dead, and sometimes I said that. But it wasn’t like I was suicidal. I just thought, ‘wow, that might be the only way actually to get some rest; can’t work a dead person’ (Participant 9).

The mental health effects of caregiving were described as deeply intertwined with structural barriers such as gatekeeping by case coordinators, insufficient home care coverage, complex administrative demands, lack of respite, and emotional isolation. These impacts extended beyond individual well-being, influencing caregivers’ ability to sustain care and undermining the emotional climate of the entire family. Participants made it clear that, while they were resilient, they were also human, and resilience without support had limits to both their mental and physical well-being.

#### Physical Strain from Unrelenting Round-the-Clock Care

In addition to the mental and emotional toll of caregiving, many caregivers spoke of the profound effects on their physical health. These impacts were not incidental. They were an accepted and often unavoidable consequence of meeting their child's needs without adequate support. Parents described sacrificing basic bodily needs, such as sleep, hydration, and rest, not out of neglect but out of necessity. The immediacy of their child's care left little room for tending to their own physical health needs.

Several caregivers shared how even routine self-care tasks, such as drinking water or using the bathroom, had to be carefully planned or deferred depending on whether someone was available to supervise their child. One caregiver said, “*without backup, basic needs are deprioritized*” (Participant 3). Others described the cumulative impact of physically demanding caregiving tasks, such as lifting, repositioning, and transferring children into vehicles or wheelchairs. While many were aware of the long-term damage these activities were causing to their bodies, they saw few alternatives. As one caregiver reflected:

It's not great, my health. I am in my 30 s, and I likely have the body of a 60-year-old, and it's not good. I recognize that what I’m doing is not good, but… what should I do? It's either ignore my health and concentrate on my son's health or ignore his health to concentrate on mine… You can’t do both together (Participant 12).

This sense of trade-off between the needs of their child versus their own health was a recurring theme. Caregivers did not describe themselves as unaware of the consequences but rather as trapped within a system that forced them to choose between their well-being and their child's safety.

Sleep deprivation was a particularly intense and widespread concern. Many caregivers reported months or years without uninterrupted sleep, especially in the early stages following diagnosis or during periods of medical instability. One parent recounted their breaking point:

For the first nine months, there was really no help. I was basically up with him every night, watching him for [his medical condition symptoms] and doing everything… I broke down and I said… ‘I do need to sleep at some point. It's been nine months and I’m tired.’ I just lost it… But chronic sleep deprivation will do that to you (Participant 9).

Even for those with some caretaking support in place, restful sleep remained elusive. Many described being in a state of hypervigilance, waking to every noise, movement, or change in breathing. This constant alertness often led to physical exhaustion:

I’m hypervigilant, so I don’t get a full, restful sleep. And every little noise will wake me up… I’m not sleeping, I’ll doze off and on all night (Participant 6).

For some (n = 6), the unrelenting nature of these routines led to a deep sense of futility and exhaustion. One caregiver poignantly captured this through metaphor:

It's like rolling the boulder up the hill to just have it roll to the bottom of the hill again the next morning (Participant 5).

Parents acknowledged that neglecting their own health was not sustainable, such as:

There are definitely days where I realize at like 2 PM that I haven’t peed. Like I’ve just been holding it all day. There are days I don’t drink enough water because I know I won’t have time to pee if I do (Participant 5).

Yet, the caregivers felt they had no realistic alternatives. As care demands continued, some began to question how long they could maintain this pace, especially without affordable, formalized and high-quality caregiving support.

#### Financial Pressures of Unpaid Care

The social, emotional, and physical demands of caregiving were compounded by a persistent and often overwhelming financial strain. Caregivers repeatedly emphasized that caregiving responsibilities were incompatible with traditional employment structures, forcing many to reduce work hours, accept demotions, or leave the workforce altogether. Even when employers offered flexibility, the demands of 24/7 caregiving, such as appointments, emergencies, equipment management, and administrative advocacy, frequently exceeded what could be accommodated. One caregiver expressed the career-altering impact of caregiving and the absence of societal or structural support:

I can’t think of one example of someone I’ve met who has managed to keep their full-time job at the same pay rate and maintain the same advancement… I’ve lost my career. I choose to give it up because of the stress of trying to be a great worker and a great [caregiver] (Participant 9).

Participants did not describe these decisions as voluntary career pivots, but as survival strategies in response to the inflexibility of employment systems and the unpredictability of their child's care needs. The shift from being a paid worker to a full-time, unpaid caregiver was experienced as an economic transformation, often accompanied by a loss of financial autonomy.

Several parents highlighted the paradox of performing labour-intensive care without compensation, while still facing the same, if not greater, expectations of productivity and resilience. One parent captured this reality: “*It's not like you can take a break. You're constantly in survival mode. You're doing everything yourself because you have no choice*” (Participant 14).

In contrast to institutional caregiving roles, parenting a CMC had no formal protections, scheduled time off, or compensation. The financial strain worsened due to ongoing out-of-pocket costs for medical supplies, therapy, equipment, and travel. While some supports were available, they were often insufficient, inconsistent, or failed to cover the full scope of need. One caregiver captured the balancing act of caregiving with no income and no external help:

We don’t get paid for being our child's primary caregiver. We can’t afford private care, so we do it all ourselves. We have no income, and our financial situation is constantly in crisis (Participant 5).

Despite the intense demands of caregiving, caregivers felt trapped in a cycle with no viable alternatives. As care responsibilities intensify, many wonder how long they can continue at this pace, especially without consistent, affordable and formal caregiving support. These interconnected challenges, including social isolation, identity loss, declining health, and financial strain, show how caring for children with medical complexity can affect every part of caregivers’ lives and may undermine the sustainability of home-based care without adequate systemic support.

## Discussion

This study explored the lived experiences of caregivers of CMC, offering an in-depth understanding of how intensive care is sustained within an emotionally taxing and structurally fragmented system. Our findings reveal the extensive scope of caregiving responsibilities, with participants describing overwhelming daily medical tasks alongside substantial time spent on paperwork and advocacy. This shows how caregiving goes beyond typical parenting. We also found that these demands affect every part of caregivers’ lives, including social connections, sense of self, health, and finances, showing how deeply caregiving can impact families in ways that have not been fully explored in past research. These findings align with existing literature, which indicates that caregivers often assume roles beyond traditional parenting, including administering medical treatments and managing complex care needs, without adequate training or support (Foster et al., 2022; Woodgate et al., 2023). Caring for children with medical complexity is a long-term, demanding role that significantly impacts caregivers’ physical and mental health. The boundary between “parent” and “care provider” becomes increasingly blurred as parents assume clinical tasks, such as suctioning, administering medication, and monitoring vital signs. This role blurring contributes to chronic stress, emotional exhaustion, and identity loss (Patty et al., 2024; Teicher et al., 2023), disrupting parent-child relationships and leading to unsustainable caregiving dynamics (McLorie et al., 2023). Time and energy devoted to CMC often come at the expense of partners and other children, undermining long-term family health and affecting siblings’ well-being through both the impact of illness and reduced parental availability (Chu et al., 2022; Baker & Claridge, 2023; Danford et al., 2024; Tay et al., 2021, 2024).

The role conflicts and caregiving burden are intensified by fragmented healthcare systems where caregivers must navigate bureaucratic barriers, misinformation, and shifting eligibility criteria to access entitled services (Schnell et al., 2025). Barriers are especially pronounced in home and community care, where limited provider awareness of CMC needs complicates access, and families manage hospital-to-home transitions with minimal support through uncoordinated services (Hirt et al., 2023; Teicher et al., 2023). Inadequate infrastructure for home nursing, medical equipment, and caregiver training, combined with inconsistent eligibility criteria across jurisdictions, makes access particularly difficult (Maynard et al., 2019). While our English-speaking sample limits insights into language barriers, existing literature indicates these challenges are often greater for newcomers and families who do not speak English (Cady & Belew, 2017; Cousino & Hazen, 2013; Salami & Alhalal, 2024). This forces parents into relentless advocacy, not empowerment, but exhausting compensatory labour for system failures.

Research consistently shows that caregivers of CMC report significantly higher stress and burnout levels than those caring for healthy children (Nathwani et al., 2024). With the growing shift towards home-based care, families should not assume this responsibility without adequate support from the system. System-level strategies are needed, including embedding caregiver mental health screening into routine pediatric complex care visits with streamlined referral pathways, and integrating supports within existing care pathways through virtual delivery or targeted interventions to enhance feasibility and uptake (Foster et al., 2024; Foster et al., 2023; Schnell et al., 2025). Integrated care models, such as the “medical home” and the “whole systems approach,” linking health, education, and social services, are crucial for dismantling silos and delivering coordinated, family-centred care (Pordes et al., 2018; McLorie et al., 2023).

Beyond physical and administrative demands, caregiving for CMC creates significant identity shifts as parents feel disconnected from former selves while struggling to adopt new caregiver roles. This liminal state gives rise to “identity discrepancy burden,” marked by emotional distress and loss of self (Patty et al., 2024; Woodgate et al., 2023). While Ontario has introduced programs such as Special Services at Home and Enhanced Respite, these fall short in providing timely and reliable support for medically complex care and lack formal structures supporting caregiver transition to personal or professional recovery (Foster et al., 2022; Nathwani et al., 2024). Support systems must acknowledge the evolving caregiver identity and offer structured interventions, such as peer mentoring, caregiver coaching, or reintegration support (Foster et al., 2022; Foster et al., 2023).

While many participants expressed adaptation and commitment, few identified with the discourse of “resilience” (i.e., the capacity to sustain well-being and adapt in the face of ongoing stress and caregiving demands). Instead, they spoke of learning how to function within the context of systemic neglect, emotional exhaustion, and the ethical demands of caregiving. This finding aligns with critiques of resilience narratives, suggesting resilience places the onus of adaptation on individuals while obscuring the structural inequities that create the barriers they need to overcome to care for their child (Foster et al., 2022; Schnell et al., 2025). Our data advocates for a shift from individualized interventions to more collective, relational, and structural solutions to increase a caregiver's resilience. These include sustained investments in home care nursing, integrating mental health services, and co-designing care systems in partnership with caregivers.

### Limitations, Implications, and Recommendations

This study provides a rich, contextualized account of how caregivers experience and sustain caregiving for CMCs. However, several interpretive and methodological limitations warrant attention. The sample consisted primarily of English-speaking mothers already engaged with formal pediatric complex care services, which may not fully represent the perspectives of caregivers who are less connected, structurally marginalized, or navigating care more informally. While some demographic variation was present (e.g., income levels and service access), having more variability of the caregiver status (i.e., fathers, co-parents, and kinship caregivers) would aid in the transferability of these findings across other caregiving groups, household structures, and cultural contexts. Additionally, data were collected at a single point in time and may reflect moments of distress, adaptation, or resilience. Future research could investigate how meaning-making processes and experiences of uncertainty differ across various caregiving contexts or stages. As with all reflexive thematic analysis, findings are shaped by the researchers’ professional and personal positioning. Future work should consider participatory approaches that involve caregivers as co-analysts and/or collaborators. Research must also center the experiences of racialized, low-income, rural, and linguistically diverse families, and adopt intersectional, family-based designs that account for how caregiving is distributed, constrained, and sustained across different social contexts and systems of care. Practice-level interventions could include adding routine caregiver mental health screening to pediatric complex care visits, standardizing eligibility criteria, enhancing case coordinator training to improve service access and ensure accurate information dissemination, and developing reliable respite services to fill persistent gaps in formal support. Educational strategies could train providers to recognize the impacts of caregiver burden and identity loss, establish peer mentorship programs that build on informal networks identified by participants, and offer structured advocacy training to reduce the exhausting work families undertake to navigate fragmented systems.

## Conclusion

The caregiving experience for a child with medical complexity has profound and lasting effects on the social functioning, identity development, and well-being of caregivers. The absence of consistent home care, emotional support, financial protections, and system responsiveness places families in a sustained state of strain and vulnerability. Addressing these challenges requires the development of comprehensive, accessible, and relationally informed policies and services that support both the child and the caregiver simultaneously, rather than requiring a choice between them. Investing in systemic and integrated mental health services, reliable respite care, income support, and caregiver-informed care models are essential to ensuring that families are not left to bear the weight of caregiving alone.

## Supplemental Material

sj-docx-1-cjn-10.1177_08445621251380909 - Supplemental material for “Rolling the Boulder Up the Hill”: A Qualitative Study of Parents’ Experiences Providing Ongoing Care at Home for Their Child with Complex Medical Needs Supplemental material, sj-docx-1-cjn-10.1177_08445621251380909 for “Rolling the Boulder Up the Hill”: A Qualitative Study of Parents’ Experiences Providing Ongoing Care at Home for Their Child with Complex Medical Needs by Joanne Tay, Adam Rapoport, Jamie Crawley, Joanne Ta and Jessica C. Kichler in Canadian Journal of Nursing Research
