# Supplementary material for: “Rolling the Boulder Up the Hill”: A Qualitative Study of Parents’ Experiences Providing Ongoing Care at Home for Their Child with Complex Medical Needs
Source: Can J Nurs Res. 2025 Oct 16;58(1):18–29. doi: 10.1177/08445621251380909 (PMC12789259; doi:10.1177/08445621251380909)
Supplement: sj-docx-1-cjn-10.1177_08445621251380909 - Supplemental material for “Rolling the Boulder Up the Hill”: A Qualitative Study of Parents’ Experiences Providing Ongoing Care at Home for Their Child with Complex Medical Needs [file sj-docx-1-cjn-10.1177_08445621251380909.docx]

**Supplementary Table 1.**

*Semi-Structured Interview Guide*

| **No.** | **Questions** |
| --- | --- |
| 1 | Tell me a bit about your role as your child’s primary caregiver and the needs or support your child requires at home? |
| 2 | As a parent/primary caregiver, what daily supports do you provide your child with at home? |
| 4 | What was it like accessing home care services? |
| 5 | Can you share your experience (either positive or negative) working with healthcare providers who provide these home care services? |
